# A novel mycobacterial Hsp70-containing fusion protein targeting mesothelin augments antitumor immunity and prolongs survival in murine models of ovarian cancer and mesothelioma

**DOI:** 10.1186/1756-8722-7-15

**Published:** 2014-02-24

**Authors:** Jianping Yuan, Satoshi Kashiwagi, Patrick Reeves, Jean Nezivar, Yuan Yang, Nadiah Hashim Arrifin, Mai Nguyen, Gilberte Jean-Mary, Xiaoyun Tong, Paramjit Uppal, Svetlana Korochkina, Ben Forbes, Tao Chen, Elda Righi, Roderick Bronson, Huabiao Chen, Sandra Orsulic, Timothy Brauns, Pierre Leblanc, Nathalie Scholler, Glenn Dranoff, Jeffrey Gelfand, Mark C Poznansky

**Affiliations:** 1 Vaccine and Immunotherapy Center, Division of Infectious Diseases, Department of Medicine, Massachusetts General Hospital, 149 13th Street, Charlestown, Boston, MA 02129, USA; 2 Department of Pathology, Harvard Medical School, Boston, USA; 3 Ragon Institute of MGH, MIT and Harvard University, Boston, USA; 4 Women’s Cancer Research Institute, Cedars-Sinai Medical Center, Los Angeles, USA; 5 Penn Ovarian Cancer Research Center, Department of Obstetrics and Gynecology, University of Pennsylvania, Philadelphia, USA; 6 Department of Medical Oncology and Cancer Vaccine Center, Dana-Farber Cancer Institute, Boston, USA; 7 Department of Medicine, Brigham and Women’s Hospital, Harvard Medical School, Boston, USA

**Keywords:** Mycobacterial Hsp70, Mesothelin, Single chain variable fragment, Cancer immunotherapy, Murine tumor model

## Abstract

**Background:**

Although dendritic cell (DC) vaccines are considered to be promising treatments for advanced cancer, their production and administration is costly and labor-intensive. We developed a novel immunotherapeutic agent that links a single-chain antibody variable fragment (scFv) targeting mesothelin (MSLN), which is overexpressed on ovarian cancer and mesothelioma cells, to *Mycobacterium tuberculosis* (MTB) heat shock protein 70 (Hsp70), which is a potent immune activator that stimulates monocytes and DCs, enhances DC aggregation and maturation and improves cross-priming of T cells mediated by DCs.

**Methods:**

Binding of this fusion protein with MSLN on the surface of tumor cells was measured by flow cytometry and fluorescence microscopy. The therapeutic efficacy of this fusion protein was evaluated in syngeneic and orthotopic mouse models of papillary ovarian cancer and malignant mesothelioma. Mice received 4 intraperitoneal (i.p.) treatments with experimental or control proteins post i.p. injection of tumor cells. Ascites-free and overall survival time was measured. For the investigation of anti-tumor T-cell responses, a time-matched study was performed. Splenocytes were stimulated with peptides, and IFNγ- or Granzyme B- generating CD3^+^CD8^+^ T cells were detected by flow cytometry. To examine the role of CD8^+^ T cells in the antitumor effect, we performed *in vivo* CD8^+^ cell depletion. We further determined if the fusion protein increases DC maturation and improves antigen presentation as well as cross-presentation by DCs.

**Results:**

We demonstrated *in vitro* that the scFvMTBHsp70 fusion protein bound to the tumor cells used in this study through the interaction of scFv with MSLN on the surface of these cells, and induced maturation of bone marrow-derived DCs. Use of this bifunctional fusion protein in both mouse models significantly enhanced survival and slowed tumor growth while augmenting tumor-specific CD8^+^ T-cell dependent immune responses. We also demonstrated *in vitro* and *in vivo* that the fusion protein enhanced antigen presentation and cross-presentation by targeting tumor antigens towards DCs.

**Conclusions:**

This new cancer immunotherapy has the potential to be cost-effective and broadly applicable to tumors that overexpress mesothelin.

## Background

The goal of cancer immunotherapy is to stimulate the immune system to destroy cancer cells. Numerous strategies that involve tumor antigen-specific and non-specific activation of the immune system have been developed. These include dendritic cell (DC) vaccines, adoptive T-cell therapy and immune checkpoint blockade [1-3]. Antigen-specific active immunotherapy is expected to be the most attractive strategy because of its capacity to induce both therapeutic and protective T-cell immunity. Among various approaches, DC vaccine is considered to be a promising treatment for advanced cancer, based on the ability of DCs to orchestrate all of the elements of the immune system. DCs capture tumor antigens, process these antigens into peptides as they move to the draining secondary lymphoid organs, and present the peptides to naïve T cells, thus inducing anti-tumor cellular immune responses. DCs can also activate B cells, NK cells, and NKT cells [1]. In pre-clinical and clinical studies that exploited DCs as a means to improve vaccine efficiency, autologous DCs are loaded *ex vivo* with antigens and re-administered to the patient. For example, Sipuleucel-T (Provenge) that consists of *ex vivo* activated autologous peripheral blood mononuclear cells (PBMCs) including antigen-presenting cells (APCs), has resulted in a significant survival benefit in Phase III trials for prostate cancer [4]. However, the production and administration of these tailor-made DC vaccines are costly and labor-intensive [5].

As a next-step in the development of DC vaccines, we designed a recombinant protein that contains a *Mycobacterium tuberculosis* heat shock protein 70 (MTBHsp70) fused to a single chain variable fragment (scFv) derived from human B cells that targets mesothelin. Mesothelin (MSLN) is a validated immunotherapy target that is highly overexpressed on the surface of common epithelial cancers including ovarian cancers, epithelial malignant mesotheliomas, ductal pancreatic adenocarcinomas, and lung adenocarcinomas, while expressed at relatively low levels only in mesothelial cells lining the pleura, pericardium, and peritoneum in healthy individuals [6-9]. Several therapeutic agents targeting MSLN are evaluated in preclinical and clinical studies such as the recombinant immunotoxin SS1P [9-11]. In our fusion protein, the anti-MSLN scFv moiety was originally isolated from a yeast-display human scFv library [12] and demonstrated the ability to recognize both membrane-bound and soluble MSLNs and inhibit CA125/MSLN-dependent cell adhesion [13-15]. The recombinant MTBHsp70 protein provides immunostimulatory functions including the activation of monocytes and DCs to produce CC-chemokines that attract antigen processing and presenting DCs, macrophages, and effector T and B cells, enhanced DC aggregation and maturation [16,17], induction of the cytotoxic activity of natural killer cells [18], and improved cross-priming of T cells which is dependent on DCs [19]. The capabilities of MTBHsp70 as a potent immune adjuvant have been well characterized in cancer models including murine models of melanoma and lymphoma [18,20-24]. While in these studies, proteins or peptides fused with Hsp70 used for immunizations in mice were shown to generate humoral or cellular immune responses, we expect that fusion of anti-MSLN scFv and MTBHsp70 takes advantage of the immune-activating action of MTBHsp70 and the tumor-targeting activity of the scFv, which will yield anti-tumor responses against the broadest profile of tumor antigens.

We evaluated the therapeutic efficacy of this MSLN-targeted fusion protein in syngeneic mouse models of ovarian cancer and mesothelioma and examined its mechanism of action in *in vitro* and *in vivo* cross-presentation assay systems. These studies demonstrate that this bifunctional fusion protein significantly enhances survival and slows tumor growth through the augmentation of tumor-specific cell-mediated immune responses.

## Results

### Expression of scFvMTBHsp70 fusion protein and MTBHsp70

**Figure 1:**
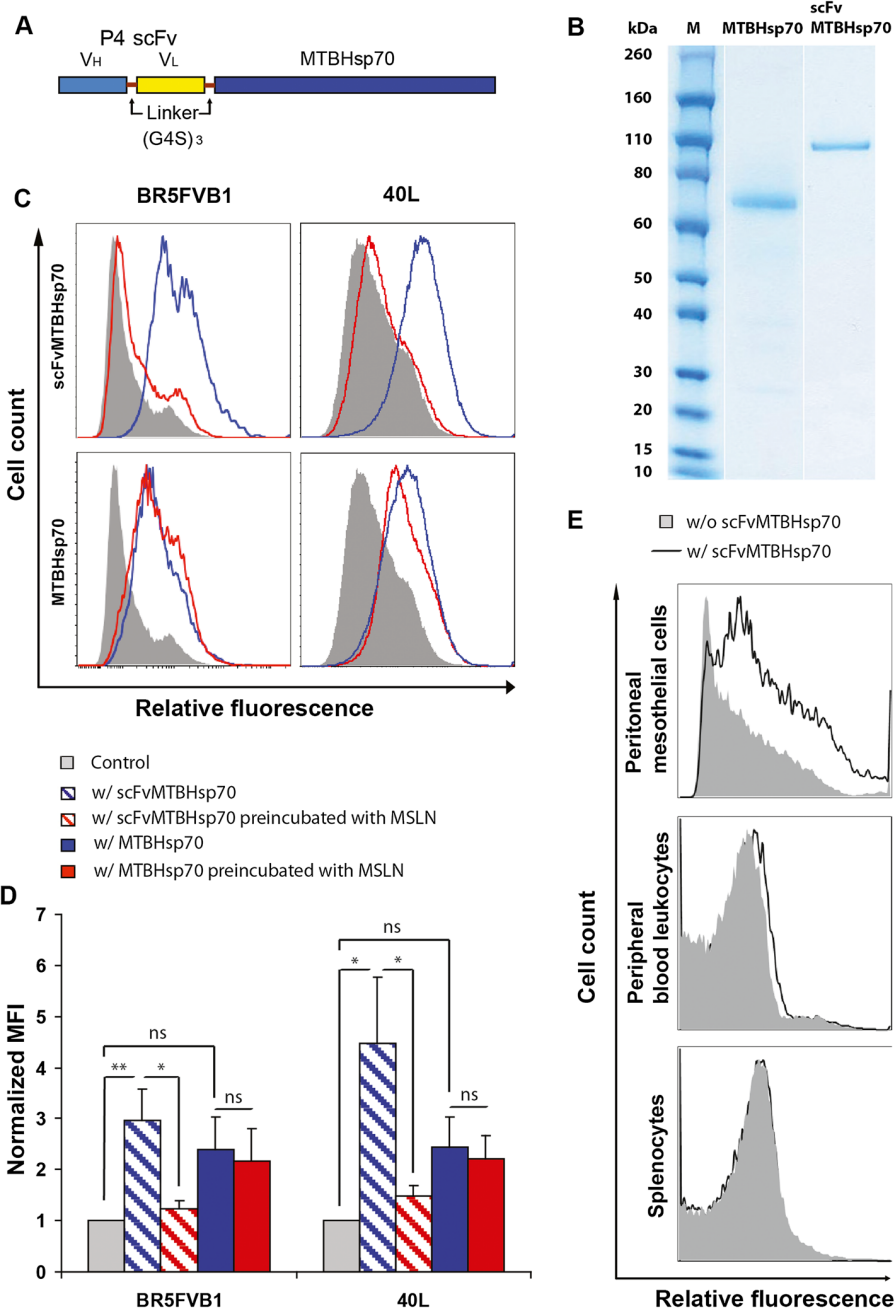
**Structure and analysis of scFvMTBHsp70 fusion protein. A**, anti-MSLN V_H_ and V_L_ are linked with a (G4S)_3_ linker and fused to full length MTBHsp70 with a (G4S)_3_ linker. **B**, RAPIDstain based on Coomassie dye following purification and hIgG-Fc tag removal of MTBHsp70 and scFvMTBHsp70. **C**, BR5FVB1 ovarian cancer cells and 40L mesothelioma cells were incubated with 40 μg/ml scFvMTBHsp70 or 26 μg/ml MTBHsp70 (blue line), or without either protein (solid), followed by anti-MTBHsp70 (IgG2a), biotinylated anti-IgG2a, and Streptavidin-APC, and then analyzed by flow cytometry. To confirm that the scFv portion of the fusion protein binds to MSLN on the surface of tumor cells, scFvMTBHsp70 or MTBHsp70 was preincubated with 12 μg/ml recombinant human MSLN for 30 min (red line) before being added to the cells. Data are representative of three independent experiments in duplicate tubes. **D**, Median fluorescence intensity (MFI) values of cells stained with scFvMTBHsp70 or MTBHsp70 normalized to cells stained without either protein. Data are expressed as means ± SEM in arbitrary units. P values were determined using One-Way ANOVA followed by Turkey’s multiple comparison tests. *,p < 0.05; **,p < 0.01;ns, non-significant. **E**, scFvMTBHsp70 binds with peritoneal mesothelial cells at a low level compared to ovarian cancer and mesothelioma cells. Binding of the fusion protein is at very low or undetectable levels on PBLs and splenocytes. Thick line, with incubation of scFvMTBHsp70; solid, without incubation of scFvMTBHsp70. Data are representative of three independent experiments.

The structure of scFvMTBHsp70 is shown in Figure 1A. V_H_ and V_L_ from anti-MSLN P4 scFv [13] are linked using a (G4S)_3_ linker and fused to full length MTBHsp70 with a (G4S)_3_ linker in between. As shown in Figure 1B, only one protein band was observed with a molecular weight of approximately 100 kDa for scFvMTBHsp70, and one protein band with a molecular weight of 70 kDa for MTBHsp70, which match the expected molecular weights of these specific proteins. Endotoxin contamination levels in scFvMTBHsp70 and MTBHsp70 were found to be very low, at less than 50 EU per mg of protein.

### scFvMTBHsp70 binds to BR5FVB1 ovarian cancer cells and 40L mesothelioma cells through the interaction of scFv with MSLN on the surface of tumor cells

Binding of scFvMTBHsp70 or MTBHsp70 to BR5FVB1 ovarian cancer cells or 40L mesothelioma cells, as determined by flow cytometry, is shown in Figure 1C and D. Binding of scFvMTBHsp70 to MSLN-expressing tumor cells was almost completely inhibited by preincubation of scFvMTBHsp70 with recombinant human MSLN. Although MTBHsp70 also binds to these MSLN-expressing tumor cells, the level of binding is not significantly different from background (p = 0.187 for BR5FVB1 cells, and p = 0.086 for 40L cells). Furthermore, the binding of MTBHsp70 to cancer cells cannot be blocked by recombinant MSLN. These data support the view that binding of scFvMTBHsp70 to these tumor cells occurred via the interaction of the scFv portion of the fusion protein with MSLN on the surface of tumor cells. Binding of these proteins with 40L mesothelioma cells was further compared using fluorescence microscopy. scFvMTBHsp70 shows significantly stronger binding intensity as compared to MTBHsp70 (Additional file 1: Figure S1A and B). In order to determine if scFvMTBHsp70 also binds to normal tissue in addition to tumor cells, we incubated the fusion protein with peripheral blood leukocytes (PBLs), splenocytes, or peritoneal mesothelial cells from healthy FVB/NJ mice, and stained the cells using the same method as was used for staining tumor cells. As shown in Figure 1E, scFvMTBHsp70 binds with peritoneal mesothelial cells at a low level compared to ovarian cancer and mesothelioma cells. Binding of the fusion protein is at very low or undetectable levels on PBLs and splenocytes. Since scFvMTBHsp70 may potentially target peritoneal mesothelial cells, we also explored whether it could induce inflammation in peritoneal mesothelial tissues. We injected naïve mice with saline, scFvMTBHsp70, or MTBHsp70 plus P4 scFv at the same doses as those used for tumor therapy described in Method, sacrificed the mice 7 days post final treatments, and examined haematoxylin and eosin (H&E) stained sections prepared from abdominal and intestinal peritoneum. Light microscopic examination revealed no evidence of inflammation and no infiltration of inflammatory cells such as macrophages or granulocytic cells around the mesothelial cells lining the abdominal and intestinal peritoneum of the actively treated or control animals. Representative microscopic images are shown in Additional file 2: Figure S2.

### scFvMTBHsp70 significantly prolongs ascites-free survival and overall survival in ovarian cancer- or mesothelioma-bearing mice

**Figure 2:**
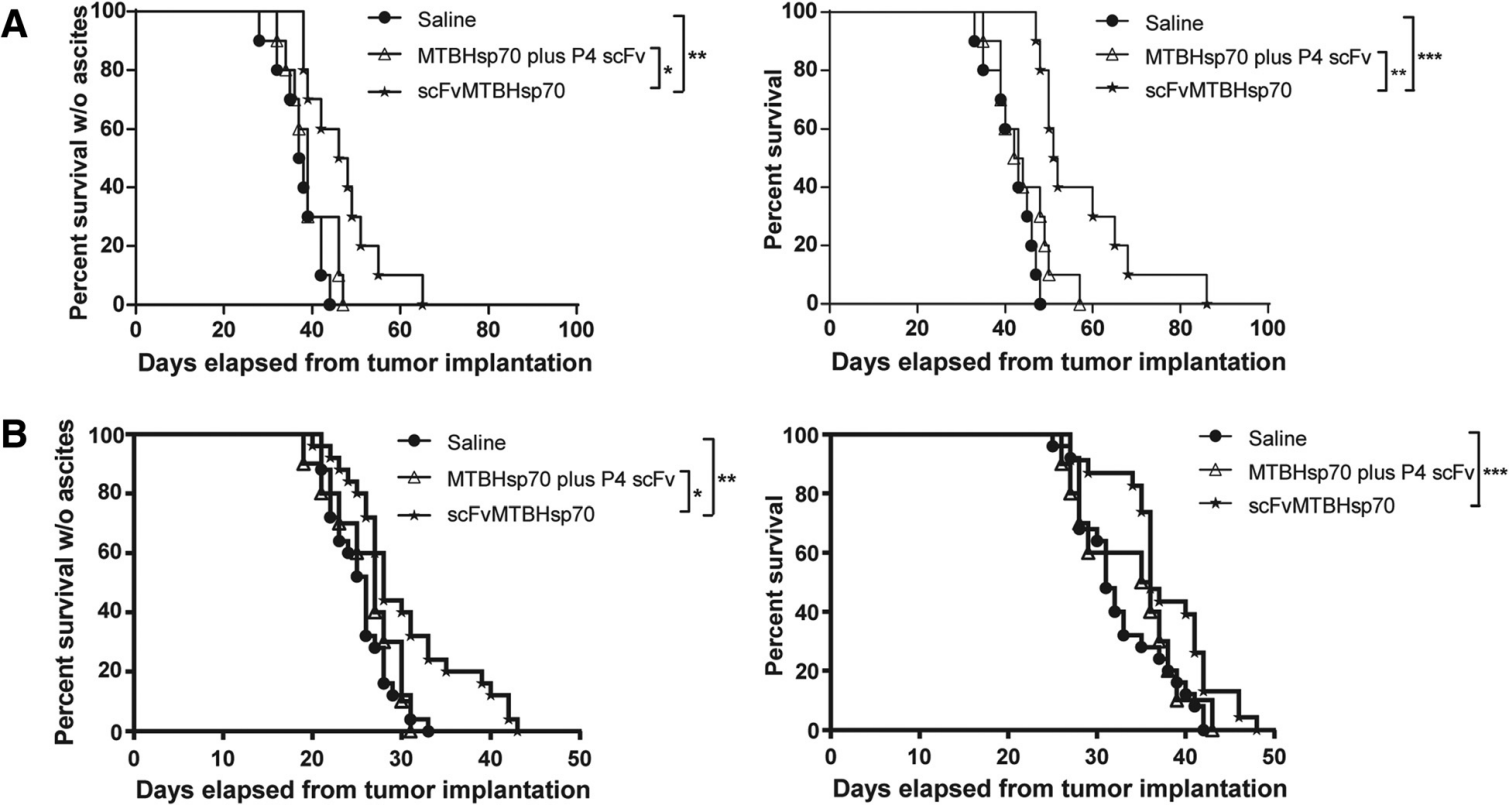
**A and B, Kaplan-Meier survival curves of tumor-bearing mice following treatment with scFvMTBHsp70, control proteins or normal saline. A**, In a syngeneic mouse model of papillary ovarian cancer in immune-competent FVB/NJ mice, scFvMTBHsp70 prolonged ascites-free survival time compared with saline (n = 10 per group, representative of two independent experiments; median survival (Med. sur.) = 47 days vs. 37.5 days), or the mixture of MTBHsp70 plus P4 scFv (Med. sur. = 39 days). scFvMTBHsp70 also prolonged overall survival time in the mice compared with saline (Med. sur. = 51.5 days vs. 43 days), or the mixture of MTBHsp70 plus P4 scFv (Med. sur. = 43 days). **B**, In a syngeneic mouse model of mesothelioma in immune-competent C57BL/6 mice, the fusion protein prolonged ascites-free survival time compared with saline-treated mice (n = 20 per group, pooled from two independent experiments; Med. sur. = 28 days vs. 26 days), or the mixture of MTBHsp70 plus P4 scFv (Med. sur. = 27 days). The fusion protein also prolonged overall survival time compared with saline (Med. sur. = 36 days vs. 31 days). P values were determined using the log-rank test. *,p < 0.05; **,p < 0.01; ***,p < 0.001.

To determine whether scFvMTBHsp70 can prolong survival in tumor-bearing mice, we first evaluated the protein in a syngeneic mouse model of papillary ovarian cancer using immune-competent FVB/NJ mice. As shown in Figure 2A, scFvMTBHsp70 prolonged both ascites-free and overall survival time compared with saline or the equimolar mixture of MTBHsp70 plus P4 scFv. To further support the efficacy of this fusion protein in prolonging survival in MSLN-expressing tumor-bearing mice, we evaluated this protein in a second syngeneic mouse model of mesothelioma using immune-competent C57BL/6 mice. Animals treated with scFvMTBHsp70 showed significantly prolonged ascites-free and overall survival time compared with saline- or MTBHsp70 plus P4 scFv- treated mice (Figure 2B).

### scFvMTBHsp70 enhances anti-tumor CD8^+^ T-cell responses in ovarian tumor-bearing mice

**Figure 3:**
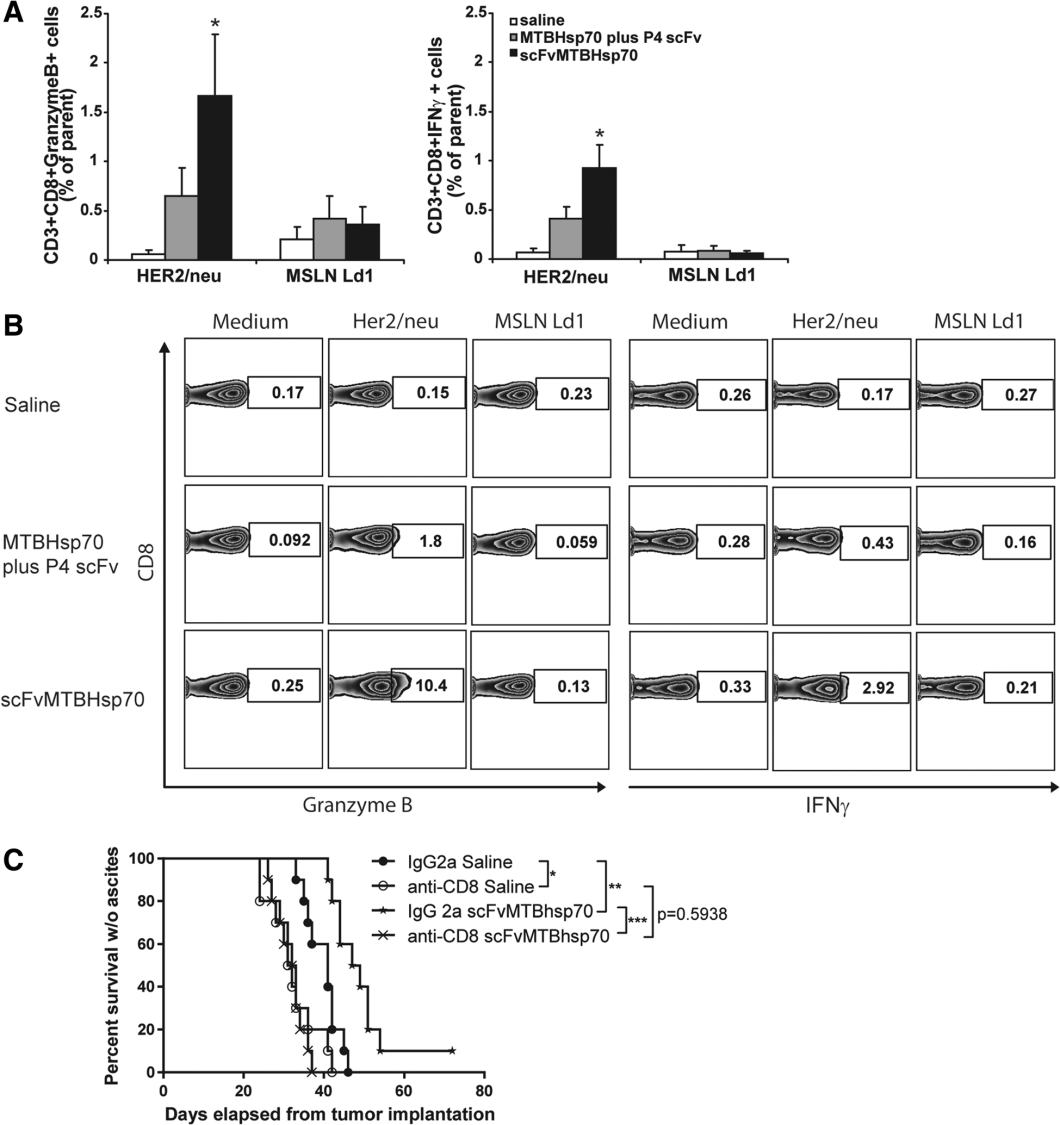
**Anti-tumor specific CD8**^**+ **^**T-cell functions in tumor-bearing mice following different treatments. A**, Splenocytes harvested from mice treated with scFvMTBHsp70 fusion protein, equimolar mixture of MTBHsp70 plus P4 scFv, or saline (n = 10 per group) were re-stimulated with Her2/neu peptide or MSLN Ld1 peptide. Results are reported as the difference between nonstimulated (media alone) and stimulated cells and expressed as the frequency of parent CD3^+^CD8^+^ cells. P values were determined using One-Way ANOVA followed by Dunnett’s multiple comparison tests. **B**, Representative flow data are presented. **C**, *In vivo* CD8^+^ T-cell depletion study. FVB/NJ mice were injected i.p. with anti-CD8 mAb or an isotype-matched irrelevant rat IgG2a, and were treated with scFvMTBHsp70 or saline as described in the methods. CD8^+^ T-cell depletion significantly and negatively impacted ascites-free survival in the scFvMTBHsp70 treated BR5FVB1 tumor-bearing animals compared to non depleted actively treated (n = 10 per group, representative of two independent experiments; Med. sur. = 32.5 days vs. 48 days) animals. After CD8^+^ T cells depletion, scFvMTBHsp70 treatment did not delay onset of disease (clinically evident ascites), compared with saline (Med. sur. = 32.5 days vs. 31.5 days; p = 0.5938). P values were determined using log-rank test. *,p< 0.05; **,p < 0.01, ***,p < 0.001.

To investigate whether the anti-tumor effects of scFvMTBHsp70 was associated with anti-tumor effector CD8^+^ T-cell responses, we re-stimulated splenocytes from ovarian tumor-bearing FVB mice that received different treatments with the CD8^+^ T-cell Her2/neu epitope or MSLN Ld1 as a negative control, *ex vivo*, and analyzed the cells for production of IFNγ and Granzyme B using flow cytometry. We previously showed that Her2/neu is expressed by BR5FVB1 cells [25]. Ld1 is an in-house designed H2^d^-restricted MSLN peptide that did not induce ovarian cancer specific T-cell response in H-2^q^ FVB mice. We demonstrated significantly greater anti-Her2/neu CD8^+^ T-cell responses in splenocytes from scFvMTBHsp70-treated mice compared to mice treated with saline or a simple mixture of MTBHsp70 plus P4 scFv, as measured by IFNγ and Granzyme B production by CD8^+^ T cells (Figure 3A and B). This indicates that scFvMTBHsp70 enhances anti-tumor specific CD8^+^ T-cell responses in ovarian tumor-bearing mice. However, no significant difference was seen in the number of tumor-infiltrating CD8^+^ T cells and no tumor-infiltrating Foxp3^+^ T cells were seen in tumors from mice in different treatment groups, indicating that scFvMTBHsp70 may improve effector cell function rather than the number of intratumoral CD8^+^ T cells (Additional file 3: Figure S3A and B).

### scFvMTBHsp70 is able to prime an adaptive, tumor-specific immune response that has an absolute requirement for tumor-specific CD8^+^ T cells

To determine whether CD8^+^ T cells play a major role in the protective anti-tumor effects observed in mice treated with scFvMTBHsp70, we conducted *in vivo* CD8^+^ T-cell depletion experiments using monoclonal antibodies. The absence of circulating CD8^+^ cells in peripheral blood, following depletion, was confirmed by flow cytometry (Additional file 4: Figure S4A and B). As shown in Figure 3C, CD8^+^ T-cell depletion significantly and negatively impacted ascites-free survival in the scFvMTBHsp70-treated BR5FVB1 tumor-bearing animals compared to non-depleted, actively-treated animals. Following CD8^+^ T-cell depletion, scFvMTBHsp70 treatment did not delay onset of disease (clinically evident ascites), compared to saline treatment. Therefore, our data suggest that the priming of an adaptive, tumor-specific immune response by scFvMTBHsp70 treatment is chiefly mediated by tumor-specific CD8^+^ T cells.

### scFvMTBHsp70 stimulates maturation of murine bone marrow-derived dendritic cells

**Figure 4:**
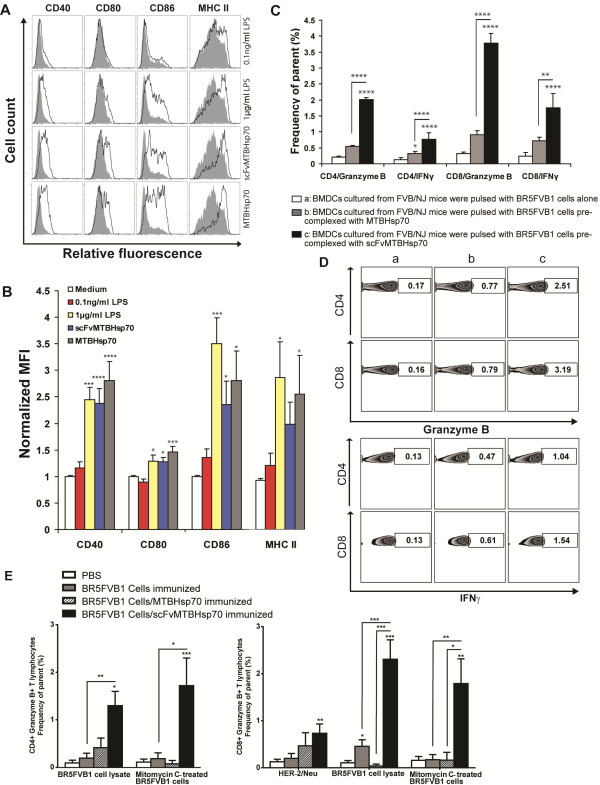
**scFvMTBHsp70 induces DC maturation and promotes antigen presentation and cross-presentation. A**, CD11c^+^ BMDCs isolated form FVB/NJ mice were incubated for 24 h with 2 μg/ml scFvMTBHsp70, 1.3 μg/ml MTBHsp70, 1 μg/ml LPS as positive control, or 0.1 ng/ml LPS as contamination control (thick lines), or medium only (solid), stained for CD11c, CD40, CD80, CD86, and MHC II, and analyzed by flow cytometry. Histograms were gated on CD11c^+^ DCs. Data are representative of three independent experiments in duplicate wells. **B**, Median fluorescence intensity (MFI) of LPS- or protein-stimulated BMDCs normalized to MFI of BMDCs maintained in medium. Data are expressed as means ± SEM in arbitrary units. P values were determined using One-Way ANOVA followed by Dunnett’s multiple comparison tests. **C**, BMDCs cultured from FVB/NJ mice were pulsed with BR5FVB1 cells alone (Column a), or BR5FVB1 cells pre-complexed with MTBHsp70 (Column b) or scFvMTBHsp70 (Column c), and then incubated with BR5FVB1 tumor cell-primed T cells. Intracellular granzyme B and IFNγ expressions in CD3^+^CD4^+^ and CD3^+^CD8^+^ T cells were analyzed by flow cytometry. Data from three independent experiments in duplicate wells are pooled and analyzed using One-Way ANOVA followed by Turkey’s multiple comparison tests. Data are presented as mean ± SEM. **D**, Representative flow data are presented. **E**, scFvMTBHsp70 enhanced tumor cell immunogenicity *in vivo.* Results are reported as the difference between nonstimulated (media alone) and stimulated cells and expressed as the frequency of parent CD3^+^CD4^+^ or CD3^+^CD8^+^ cells. P values were determined using One-Way ANOVA followed by Turkey’s multiple comparison tests. *,p < 0.05; **,p < 0.01; ***,p < 0.001; ****,p < 0.0001.

In order to investigate immunological mechanisms involved in the scFvMTBHsp70-enhanced anti-tumor immune response, we first examined if the scFvMTBHsp70 or MTBHsp70 proteins used in our study could stimulate maturation of bone marrow-derived dendritic cells (BMDCs) as shown in previous studies [16,17]. We stimulated CD11c^+^ BMDCs with 2 μg/ml of scFvMTBHsp70 or an equimolar amount of MTBHsp70 (1.3 μg/ml). 1 μg/ml lipopolysaccharide (LPS) was used as positive control. To determine whether the BMDC maturation was attributable to LPS contamination of the recombinant proteins used in this study, we also incubated BMDCs with 0.1 ng/ml LPS, which was the equivalent amount of endotoxin found in 2 μg/ml scFvMTBHsp70. After a 24 h-incubation, both scFvMTBHsp70 and MTBHsp70 induced DC maturation indicated by an increase in the expression of CD40, CD80, CD86 and MHC class II molecules in comparison to the control cultures in medium. The increased expression of these DC maturation markers were comparable to those on cells stimulated with 1 μg/ml LPS. The contamination control showed that addition of 0.1 ng/ml LPS did not replicate the effects of scFvMTBHsp70 or MTBHsp70, allowing us to discriminate the scFvMTBHsp70- or MTBHsp70-specific effects from effects of LPS (Figure 4A and B).

### The scFvMTBHsp70 fusion protein increases tumor antigen presentation and cross-presentation by DC *in vitro*

In the current study, we demonstrated that splenic CD8^+^ T cells from scFvMTBHsp70-treated tumor-bearing mice could produce cytokines upon specific tumor antigen stimulation *ex vivo*, which was associated with their antitumor therapeutic efficacy *in vivo*. To determine whether scFvMTBHsp70 promotes tumor specific T-cell responses by enhancing antigen presentation and cross-presentation by antigen presenting cells, we co-cultured BR5FVB1 tumor cell-primed T cells with DCs that had been pulsed with BR5FVB1 tumor cells in the presence of scFv-MTBHsp70, MTBHsp70, or PBS. The scFvMTBHsp70/tumor cell-pulsed DCs induced significantly higher production of IFN-γ and Granzyme B from both CD4^+^ and CD8^+^ tumor cell-primed T cells as compared with MTBHsp70 or PBS, indicating that scFvMTBHsp70 enhances tumor antigen presentation and cross-presentation by DCs (Figure 4C and D).

### scFvMTBHsp70 enhances tumor cell immunogenicity *in vivo*

Having demonstrated *in vitro* that scFvMTBHsp70 enhances tumor antigen presentation and cross-presentation by DCs, we next explored whether scFvMTBHsp70 enhances tumor antigen presentation and cross-presentation by DCs and consequently enhances tumor cell immunogenicity *in vivo*. It has been demonstrated that the high density of DCs at dermal sites facilitates the capture of tumor antigens and that local inflammation induces DC maturation and migration into draining lymph nodes, where they present antigens to naïve T cells, generating a tumor specific immune response [26]. We primed FVB mice with an intradermal (i.d.) injection of mitomycin C-treated BR5FVB1 tumor cells, followed by a booster i.d. injection of BR5FVB1 tumor cells with or without scFvMTBHsp70 or MTBhsp70. After 20 days, we dissociated skin-draining lymph nodes and re-stimulated lymph node lymphocytes with Her2/neu peptides, mitomycin C-treated BR5FVB1 tumor cells, or BR5FVB1 tumor cell lysate, and performed flow cytometric analysis for the presence of Granzyme B-generating CD4^+^ and CD8^+^ T cells. As shown in Figure 4E, we demonstrated that Granzyme B-generating CD4^+^ and CD8^+^ T cells were significantly enhanced in mice that were immunized with scFv-MTBHsp70-bound tumor cells, as compared to those in the mice immunized with tumor cells alone, MTBHsp70-bound tumor cells, or saline.

## Discussion

We have developed a novel protein-based immunotherapy consisting of a fusion of an anti-MSLN scFv of human origin and recombinant mycobacterial heat shock protein 70 that has the ability to adjuvant significant T-cell responses against specific tumor antigens. P4 scFv directed against MSLN, a surface antigen overexpressed on several types of tumor cells, is used as a means of targeting the immunotherapeutic agent. We have demonstrated that this bifunctional fusion protein effectively binds BR5FVB1 ovarian cancer cells or 40L mesothelioma cells through the interaction of scFv with MSLN on the surface of tumor cells. We found that the fusion protein significantly prolonged survival time in syngeneic mouse models of papillary ovarian cancer and malignant mesothelioma. Treatment with the fusion protein induced significant tumor-specific CD8^+^ T-cell immune responses in the splenocytes of ovarian tumor-bearing mice. Furthermore, *in vivo* CD8^+^ T-cell depletion studies demonstrated that this protective antitumor effect is mainly mediated by tumor-specific CD8^+^ T cells. Treatment using a mixture of MTBHsp70 plus P4 scFv for ovarian tumor or malignant mesothelioma-bearing mice did not increase survival or enhance tumor-specific immune responses, suggesting that only through fusion of the two elements is the immune system effectively activated. We also demonstrated that this approach does not induce inflammation in the abdominal or intestinal mesothelial tissues as a result of a bystander interaction with MSLN on normal mesothelial cells.

Several properties of MTBHsp70 appear in this study to contribute to the generation of tumor-specific CD4^+^ and CD8^+^ T-cell immune responses. First, it induces maturation of DCs. Although several previous studies suggested that MTBHsp70 had pro-inflammatory properties only when contaminated with LPS [27,28], other studies have decisively demonstrated that MTBHsp70 alone while not LPS promotes DC maturation and innate immune responses [16,17,29]. In our study, we used a fusion protein generated from a mammalian cell expression system, ensuring a minimal amount of LPS contamination. We also incubated DCs with the same amount of LPS as that found in the fusion protein and failed to replicate the effects observed with the fusion protein, supporting the view that maturation of DCs can be attributed to the fusion protein rather than LPS. Secondly, MTBHsp70 is capable of delivering epitopes for enhanced processing and MHC-I presentation by DCs to naïve CD8^+^ T cells, a process known as cross-presentation [30]. Mycobacterial Hsp70 fusion proteins have been shown to elicit both CD4+ and CD8^+^ T-cell responses although priming of CD8^+^ T cells does not appear to require CD4^+^ T cells [31,32]. We demonstrated in this study that the MSLN-targeted fusion protein elicited significant tumor-specific CD8^+^ T-cell immune responses in ovarian cancer-bearing mice, and this adaptive antitumor response has an absolute requirement for tumor-specific CD8^+^ T cells. Although at the dosing schedule used in these studies, tumor-specific T-cell responses did not eventually lead to rejection of the established tumors, they significantly prolonged survival time in tumor-bearing mice.

DCs are believed to play a pivotal role in the initiation and programming of tumor-specific T-cell responses, and are becoming an essential target in efforts to generate therapeutic immunity against cancer [33]. Two main approaches are currently under consideration for providing DCs with tumor-specific antigens. One approach is to culture patient-derived DCs *ex vivo* with an adjuvant that induces DC maturation in the presence of tumor specific antigens, followed by adoptive transfer into the patient [33]. This approach is fraught with technical and practical difficulties such as selection of a suitable antigenic target, inappropriate maturation state of selected DCs, and the difficulty of generating a sufficient number of DCs *ex vivo*. In addition, a number of investigators have recently reported that *ex vivo*-derived DC vaccines have an insignificant role in the direct priming of T cells *in vivo*[33-35].

An alternative approach to generate tumor-specific antigen bearing DCs is to induce them to take up tumor-specific antigens *in vivo*. It has been shown that *in vivo* specific targeting of tumor antigens to DCs improves the induction of antigen-specific CD4^+^ and CD8^+^ T-cell immunity. In these studies, an agonistic anti-CD40 monoclonal antibody was used to mature DCs and eliminate antigen-specific tolerance [36-39]. MTBHsp70 has also been shown to stimulate inflammation and DC maturation via an interaction with CD40 receptors on both DCs and monocytes, thus acting as an alternative ligand to CD40L [29,40]. In our study, we showed the fusion protein up-regulates surface expression of phenotypic markers of DC maturation. Interestingly, in addition to CD80, CD86, and MHC class II molecules, the expression of CD40 is also enhanced, indicating a possible positive feedback loop involving CD40 signaling components.

Beyond promoting DC maturation, the scFvMTBHsp70 fusion protein also targets tumor cells towards the matured DCs. We propose that binding of the fusion protein with both tumor cells and DCs improves phagocytosis of parts of tumor cells by DCs, and therefore any tumor antigen can be processed and loaded on both MHC class II and MHC class I molecules, and presented to CD4^+^ and CD8^+^ T cells. This could explain the observed augmentation of tumor antigen presentation and cross-presentation brought about by the fusion protein *in vitro.* This may also explain the observed increased anti-Her2/neu CD8^+^ T-cell responses in the scFvMTBHsp70-treated ovarian tumor bearing mice, although Her2/neu is not directly targeted. We recapitulated these *in vitro* findings in an *in vivo* tumor cell immunogenicity study. We used the fusion protein to activate and mature DCs in the skin such as Langerhans cells. These DCs then captured tumor cells or tumor cell fragments through the connection established by the fusion protein, and migrated to the draining lymphoid organs where they presented tumor antigens to naïve T cells. T cells recovered from the draining lymph node showed significantly enhanced responses to stimulation with a range of tumor antigens.

## Conclusion

**Figure 5:**
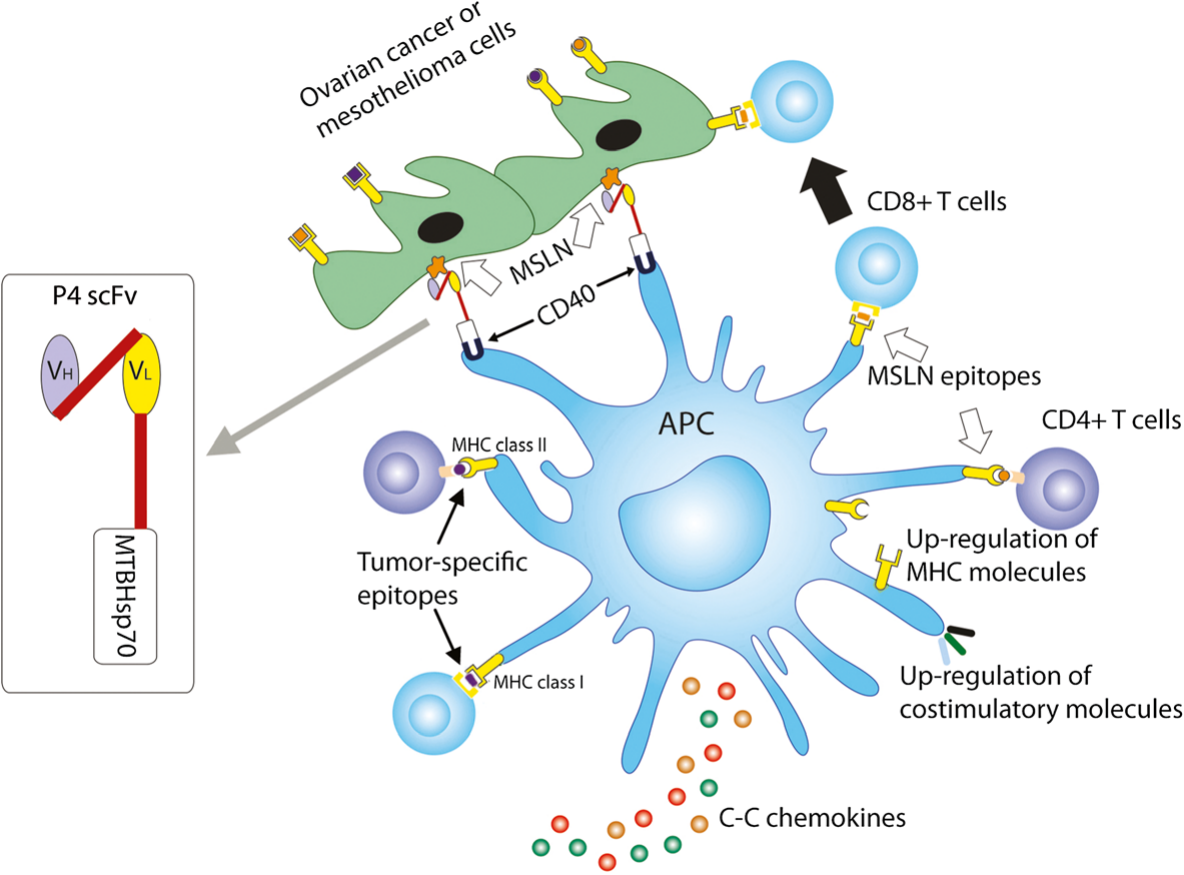
**A schematic model showing that the scFvMTBHsp70 fusion protein binds with MSLN on tumor cells and activates antigen presenting cells (APCs), thus promoting uptake of tumor cells or tumor cell fragments, and promoting tumor antigen presentation and cross-presentation as well as adjuvanting tumor specific CD4**^**+ **^**and CD8**^**+ **^**T-cell responses.**

Our study provides preclinical evidence that supports a protein-based immunotherapy that induces anti-tumor immune responses, which normally require dendritic cell-based approaches. The MSLN-targeted MTBHsp70 fusion protein binds MSLN on tumor cells, recruits and activates APCs including DCs, loads DCs *in vivo* with the broadest profile of naturally processed tumor antigens, promotes tumor antigen presentation and cross-presentation, and enhances tumor specific CD4^+^ and CD8^+^ T-cell responses (Figure 5). Our study supports the continued exploration of this novel fusion protein alone or in combination with immune checkpoint inhibitors, following conventional surgical reduction and chemotherapy for MSLN-expressing cancers. This new approach could significantly increase time to recurrence and survival in humans with ovarian cancer and mesothelioma where effective second line treatment options are very limited.

## Methods

### Production of proteins

The plasmid pQE30-MTBhsp70 that encodes full length MTBHsp70 was a generous gift from Dr. Peter Sveshnikov (Moscow Medical Academy, Russia). The plasmid pTOR2-scFv that encodes an scFv fragment specific to MSLN and the recombinant P4 scFv protein [13], generated and purified from yeast, were generous gifts from Dr. Nathalie Scholler (Penn Ovarian Cancer Research Center, University of Pennsylvania). The DNA fragment corresponding to a 15 amino acid linker (GGGGSGGGGSGGGGS) was connected to the scFv at its C-terminal using an overlap PCR approach. The PCR product scFv-linker was subcloned into pQE30-MTBhsp70 at the N-terminal of MTBhsp70. The DNA fragment for scFvMTBhsp70 was PCR amplified and cloned into pPMY5 (Promab), downstream of a human IgG1 Fc domain and separated from the Fc region by the signal cleavage sequence for Tobacco Etch Virus protease (TEV enzyme). scFvMTBHsp70, the MSLN-targeted fusion protein, was generated from HEK293 cells and purified using Protein G resin (Pierce). The Fc region of the Protein G eluted protein was then cleaved from the fusion protein by TEV enzyme (Promab) digestion. MTBHsp70 was generated using the same expression system. The production and purification of these two proteins was accomplished by Promab Biotechnologies, Inc. at Richmond CA. After purification and hIgG-Fc tag removal, the integrity of scFv-MTBHsp70 and MTBHsp70 were determined by SDS-PAGE, followed by staining with RAPIDstain (G-Bioscience). Endotoxin contamination levels in scFvMTBHsp70 and MTBHsp70 were determined by Limulus Amebocyte Lysate Assay (LAL-assay, Cambrex).

### Cells

The BR5FVB1 ovarian cancer cells, a kind gift from Dr. Orsulic in Women’s Cancer Research Institute at Cedars-Sinai Medical Center [41], or 40L mesothelioma cells, a kind gift from Dr. Kane in Department of Pathology and Laboratory Medicine at Brown University [42], were maintained at 37°C in DMEM with 2 mmol/L L-glutamine, 10 units/ml penicillin, 10 μg/ml streptomycin, and 10% fetal bovine serum in humidified atmosphere with 5% CO_2_. Cells were cultured until 80% confluent, and harvested with enzyme-free cell-dissociation buffer (Gibco) for *in vitro* tumor cell binding assays and cross-presentation studies, or harvested with Trypsin EDTA (Mediatech) for animal injections. Mouse PBLs were obtained from FVB mice via tail vein bleeds, after lysis of erythrocytes using M-lyse buffer (R&D systems). Small pieces of parietal peritoneal membrane were taken from the mice and digested in enzyme-free cell-dissociation buffer to obtain mouse peritoneal mesothelial cells. To test whether scFvMTBHsp70 or MTBHsp70 binds to the MSLN-expressing tumor cells, or non-cancerous cells, we incubated BR5FVB1 ovarian tumor cells, 40L mesothelioma cells, or normal cells from FVB mice including PBLs, splenocytes, and peritoneal mesothelial cells with 40 μg/ml scFvMTBHsp70 or 26 μg/ml MTBHsp70, followed by anti-MTBHsp70 (IgG2a) (Biodesign International), biotinylated anti-IgG2a (BD Bioscience), and Streptavidin-APC (BioLegend), and then analyzed the tumor cells by flow cytometry. As controls, cells were incubated with the reagents described above except scFvMTBHsp70 or MTBHsp70. To confirm that scFv portion of the fusion protein binds to MSLN on the surface of tumor cells, scFvMTBHsp70 or MTBHsp70 was preincubated with 12 μg/ml of recombinant human MSLN (R&D Systems) for 30 min before adding to the cells. For fluorescence microscopy, cells were cultured on coverslips until 50% confluent, stained with 10 μg/ml scFvMTBHsp70 or 6.5 μg/ml MTBHsp70, followed by mouse anti-MTBHsp70 (1:500 dilution), and Donkey anti-mouse Alexa Fluor 594 (Invitrogen, 1:500 dilution). Cells were observed using a Nikon Eclipse TiE fluorescence microscope. In some experiments, tumor cells were treated with 20 μg/ml mitomycin C at a concentration of 5 × 10^6^/ml for 1 h in a 37°C water bath, and washed with complete medium at least 3 times before use.

### Animal models and tumor treatment

Ovarian cancer was established by i.p. injection of syngeneic cancer cells BR5FVB1 (10^7^ cells per mouse) into 6-week-old female FVB/NJ mice as previously described [25]. All mice were purchased from Jackson laboratories. Intraperitoneal mesotheliomas were established by i.p. injection of syngeneic 40L cells (2 × 10^6^ per mouse) into 6-week-old male C57BL/6 mice as previously described [42]. Mice with ovarian tumors were treated 7 days after BR5FVB1 tumor cell inoculation with i.p. injections of scFvMTBHsp70 (2 μg per mouse), normal saline, or an equimolar mixture of MTBHsp70 plus P4 scFv. This was followed by 3 further treatments at 4-day intervals. In the mesothelioma model, C57BL/6 mice were treated 5 days after 40L tumor cell inoculation, and injected i.p. with scFvMTBHsp70 (2 μg per mouse), normal saline, or an equimolar mixture of MTBHsp70 plus P4 scFv. Three subsequent doses were administered at 3-day intervals, thereafter. For survival studies, we observed the mice daily 3 weeks after inoculation of BR5FVB1 cells or 1 week after inoculation of 40L cells. Tumor generations were consistently first evident via abdominal distension secondary to malignant ascites, and tumor-bearing mice were euthanized at the endpoint when there were signs of distress, including fur ruffling, rapid respiratory rate, hunched posture, reduced activity, and progressive ascites formation as previously described [25]. For the investigation of anti-tumor T-cell responses, all ovarian tumor-bearing mice were sacrificed 7 days after the final scheduled treatment. All studies were performed in a manner that was blinded to the observer under protocols that were approved by the Massachusetts General Hospital Subcommittee on Research Animal Care (SRAC).

### Treatment of naïve mice with experimental or control protein

6-week-old male C57BL/6 mice were injected i.p. with scFvMTBHsp70 (2 μg per mouse), normal saline, or an equimolar mixture of MTBHsp70 plus P4 scFv. Three subsequent doses were administered at 3-day intervals thereafter. Seven days post the administration of the final treatment, mice were sacrificed and abdominal wall and intestine were retrieved for histopathological studies of mesothelial tissues.

### *Ex vivo* assessment of tumor specific T-cell functions

Single cell suspensions were prepared from spleens. Cells were plated in round-bottomed 96-well plates, pulsed with a validated CD8^+^ T-cell Her2/neu peptide (PDSLRDLSVF, 1 μg/ml; EZBiolab) [25,43], an in-house designed H2^d^-restricted MSLN Ld1 peptide (IPLSYLCDF, 1 μg/ml; EZBiolab) that did not induce ovarian cancer specific T-cell response in H-2^q^ FVB mice, or medium alone for 72 hours when Golgi Plug (BD Bioscience) was added for the last 5 hours as previously described [44], and then stained with fluorophore-conjugated anti-CD3, anti-CD4, anti-CD8, anti-IFNγ (BD Pharmingen), and anti-Granzyme B (eBioscience) antibodies. Cells were then analyzed on a LSRII 4 laser (BD Biosciences).

### Depletion of CD8^+^ T cells *in vivo*

FVB/NJ mice were injected i.p. with 200 μg of anti-CD8 monoclonal antibody (mAb)(53–6.72, Bio X Cell) or an isotype-matched irrelevant rat IgG2a (2A3, Bio X Cell) 2 days before, 1 day before and 1 day after i.p. inoculation with BR5FVB1 ovarian tumor cells. Depletion was continued once every week until 29 days after tumor inoculation. The mice were treated with scFvMTBHsp70 or saline as described above. All the mice were bled from the tail vein and the depletion of CD8^+^ cells was examined by flow cytometry analysis of peripheral blood cells stained with fluorophore-conjugated anti-CD8 on days 7 and 28 after tumor inoculation.

### Generation and purification of bone marrow-derived DCs (BMDCs)

CD11c^+^ DCs were generated from bone marrow cells of FVB/NJ mice as described [45-47], with minor modifications. Briefly, erythrocyte-depleted mouse bone marrow cells from flushed marrow cavities were cultured in complete RPMI 1640 with 10 ng/ml GM-CSF and 1 ng/ml IL-4 at 1 × 10^6^ cells/ml. Medium was changed on day 3. On day 7, DCs were harvested by gentle pipetting and purified with magnetic microbeads conjugated to a monoclonal antibody against CD11c (MiltenyiBiotec) as described [46,48], according to the manufacturer’s recommended protocol.

### *In vitro* activation of BMDCs

CD11c^+^ BMDCs were plated in a 24-well plate at a density of 2 × 10^6^ cells/ml and incubated with 2 μg/ml scFvMTBHsp70 (105 kDa), 1.3 μg/ml MTBHsp70 (70 kDa), 1 μg/ml LPS equivalent to 10^3^ EU/ml endotoxin (InvivoGen, San Diego, CA), or 0.1 ng/ml (0.1 EU/ml) LPS equivalent to endotoxin found in 2 μg/ml of proteins (since LPS level is less than 50 EU per mg of protein) for 24 h at 37°C in humidified atmosphere with 5% CO_2_. Cells were then placed on ice, collected by vigorous pipetting, washed and stained with the following fluorophore-conjugated antibodies: anti-CD11c and anti-CD40 (eBioscience), anti-CD80 (BD Horizon), anti-CD86 and anti-MHC class II (I-A^q^) (BD Pharmingen). Afterwards, the cells were analyzed on an LSRII 4 laser (BD Biosciences).

### *In vitro* tumor antigen presentation assay

BR5FVB1 cells were harvested and treated with mitomycin C, and plated in a 96-well round-bottomed plate with 20 μg/ml scFvMTBHsp70 or 13 μg/ml MTBHsp70. After pre-incubation at 4°C for 1 h, CD11c^+^ BMDCs (ratio of tumor cells: DCs = 3: 1) were added to the wells and the plate was incubated at 37°C for 24 h. For generation of BR5FVB1 cell-primed T cells, we inoculated FVB/NJ mice by i.p. injection with 10^7^mitomycin C-treated BR5FVB1 cells and sacrificed the mice 60 days after the immunization according to the approved animal protocol. Splenocytes were then harvested, and T cells were isolated using the Pan T-Cell Isolation Kit II (MiltenyiBiotec). BR5FVB1 cell-primed T cells were then added to the wells at a DC/T-cell ratio of 1:20. After a 24-hour co-culture of BR5FVB1 cell-pulsed DCs with BR5FVB1 cell-primed T cells, the cells were harvested, washed and resuspended in PBS with 5% FBS, stained for CD3, CD4, CD8 and IFNγ, and analyzed on a LSRII 4 laser (BD Biosciences).

### *In vivo* immunization with mitomycin C-treated ovarian tumor cells

BR5FVB1 ovarian tumor cells were harvested with enzyme-free cell-dissociation buffer and treated with mitomycin C as described above. Cells were then pre-incubated with scFvMTBHsp70 (10 μg/10^6^ cells), MTBHsp70 (6.5 μg/10^6^ cells), or PBS alone at 4°C for 1 h. 6-week-old FVB mice were shaved and depilated on both left and right flanks, and then injected i.d. with 50 μl of PBS, or 1 × 10^6^ tumor cells in 50 μl of PBS with or without a pre-incubation with scFvMTBHsp70 or MTBHsp70 at both flanks.

### Histopathology

Abdominal walls and intestines from mice were fixed for at least 24 h in PBS-buffered 10% formalin. Tissues were routinely embedded in paraffin. 5 μm thick sections were stained routinely with H&E. For staining tumor-infiltrating T cells, mice were perfused with 4% paraformaldehyde (PFA) in PBS, and tumor nodules were fixed in 4% PFA/PBS for additional 2 hours, washed and infiltrated with 30% sucrose/PBS at 4°C. 6 μm thick frozen sections were stained with rat anti-mouse CD8 (BD Biosciences, 1:100 dilution) or rat anti-mouse Foxp3 (eBioscience, 1:12 dilution), followed by polyclonal rabbit anti-rat immunoglobulin/HRP (Dako, 1:750 dilution). Signal was developed with diaminobenzidine (DAB, Dako). Images were acquired on a Zeiss Axio A1 microscope. All histopathological and immunohistochemical samples were reviewed and the quantitation of the cellular infiltrate was performed in a blinded manner to the observer.

### Statistical analysis

Statistical differences between three or more experimental groups were analyzed using One-Way ANOVA, followed by Turkey’s multiple comparison tests when mean of each group is compared with that of every other group, or followed by Dunnett’s multiple comparison tests when mean of each group is compared with that of a control group. Statistical differences between two experimental groups were analyzed using Student’s *t*-test. Survival was analyzed with the Log-rank test. Prism 6.0 software (GraphPad Software) was used for all the statistical analysis.

## Abbreviations

DC: Dendritic cell; scFv: Single-chain antibody variable fragment; MSLN: Mesothelin; MTB: Mycobacterium tuberculosis; Hsp: Heat shock protein; i.p.: Intraperitoneal; i.d.: Intradermal; BMDCs: Bone marrow-derived dendritic cells; APCs: Antigen-presenting cells; PBMCs: Peripheral blood mononuclear cells; PBLs: Peripheral blood leukocytes; LPS: Lipopolysaccharide; H&E: Haematoxylin and eosin; PFA: Paraformaldehyde; DAB: Diaminobenzidine; mAb: monoclonal antibody.

## Competing interests

The authors declare that they have no competing interests.

## Authors’ contributions

JY played a role in the design of the experiments, acquisition, analysis, and interpretation of the data, and writing the manuscript. PR, JN, YY, NHA, MN, GJ-M, XT, SK, HC, PU, BF, TC and PL participated in the performance of experiments. SK and TB were involved in design of the experiments. RB was involved in data analysis. ER was involved in setting up murine ovarian cancer model. SO provided the murine ovarian cancer model. NS provided the plasmid that encodes an scFv fragment specific to MSLN and the recombinant P4 scFv protein. GD, NS and SO gave constructive input on experimental design and data analysis. JG played a role in conception and design of the fusion protein. MP and JG were involved in the conceptualization and design of the study, analysis and interpretation of datasets and in writing the manuscript. All authors read and approved the final manuscript.

## Supplementary Material

Additional file 1: Figure S1 scFvMTBHsp70 binds to 40L mesothelioma cells. 40L cells were stained with scFvMTBHsp70 or MTBHsp70, followed by mouse anti-MTBHsp70, and Donkey anti-mouse Alexa Fluor 594. Cells were observed using a Nikon Eclipse TiE fluorescence microscope. A, Representative pictures from three independent experiments. Scale bar, 10 μm. B, Images were analyzed using the NIS-Elements AR Microscope Imaging Software. Mean Fluorescence Intensity was analyzed using ImageJ. P values were determined using One-Way ANOVA followed by Turkey’s multiple comparison tests. ****,p < 0.0001. Click here for file

Additional file 2: Figure S2 scFvMTBHsp70 or MTBHsp70 plus P4 scFv treatment does not lead to infiltration of inflammatory cells into abdominal or intestinal mesothelial tissues. Samples of abdominal wall and intestine were prepared from C57BL/6 mice that had previously received multiple i.p. injections of scFvMTBHsp70, MTBHsp70 plus P4 scFv or saline as described in the Methods section. Sections of these tissues were stained with H&E, and images were acquired on a Zeiss Axio A1 microscope. Representative images from 3 animals per treatment group are shown. No detectable level of mononuclear cell or granulocyte infiltrate within mesothelial tissues was seen in any sampled tissues. Scale bar, 20 μm. Click here for file

Additional file 3: Figure S3 scFvMTBHsp70 treatment does not affect numbers of tumor-infiltrating CD8^+^ or Foxp3^+^ T cells. (A) Representative images of intratumoral CD8^+^ and Foxp3^+^ T cells from saline (n = 3), scFvMTBHsp70 (n = 3), or MTBHsp70 plus P4 scFv (n = 3) -treated mice. Mouse spleen sections were used as positive controls: CD8^+^ and Foxp3^+^ T cells are clearly evident in the sections. Scale bar, 20 μm. (B) Numbers of CD8^+^ and Foxp3^+^ cells were quantified from 3–5 randomized fields. Click here for file

Additional file 4: Figure S4 Validation of *in vivo* depletion of CD8^+^ cells in FVB/NJ mice. Mice were injected i.p. with 200 μg of anti-CD8 mAb or an isotype-matched irrelevant rat IgG2a as described in Methods. All the mice were bled from the tail vein and the depletion of CD8^+^ cells was examined by flow cytometry analysis of peripheral blood cells stained with fluorophore-conjugated anti-CD8 on days 7 and 28 after tumor inoculation. (A) Representative results of flow analyses on 10 mice per group and reported as the percentage of CD8^+^ cells in lymphocytes. (B) CD8^+^ cells in the mice treated with isotype IgG2a or anti-CD8 mAb were compared. ***,p< 0.001. Click here for file
