# Supplementary figures and images for: A novel mycobacterial Hsp70-containing fusion protein targeting mesothelin augments antitumor immunity and prolongs survival in murine models of ovarian cancer and mesothelioma
Source: J Hematol Oncol. 2014 Feb 24;7:15. doi: 10.1186/1756-8722-7-15 (PMC3943805; doi:10.1186/1756-8722-7-15)

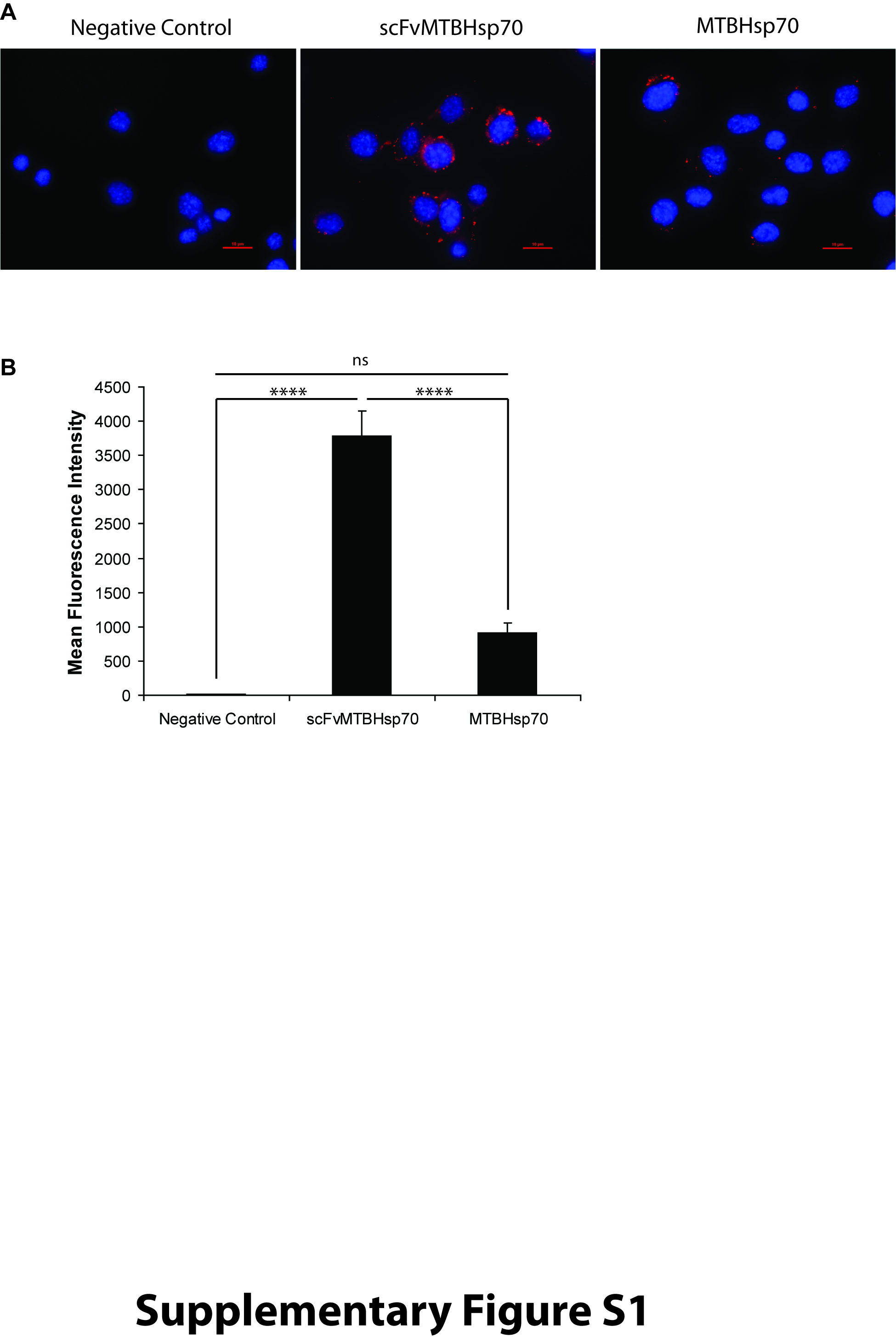

Supplement: Additional file 1: Figure S1 — scFvMTBHsp70 binds to 40L mesothelioma cells. 40L cells were stained with scFvMTBHsp70 or MTBHsp70, followed by mouse anti-MTBHsp70, and Donkey anti-mouse Alexa Fluor 594. Cells were observed using a Nikon Eclipse TiE fluorescence microscope. A, Representative pictures from three independent experiments. Scale bar, 10 μm. B, Images were analyzed using the NIS-Elements AR Microscope Imaging Software. Mean Fluorescence Intensity was analyzed using ImageJ. P values were determined using One-Way ANOVA followed by Turkey’s multiple comparison tests. ****,p < 0.0001. [file 1756-8722-7-15-S1.tiff]

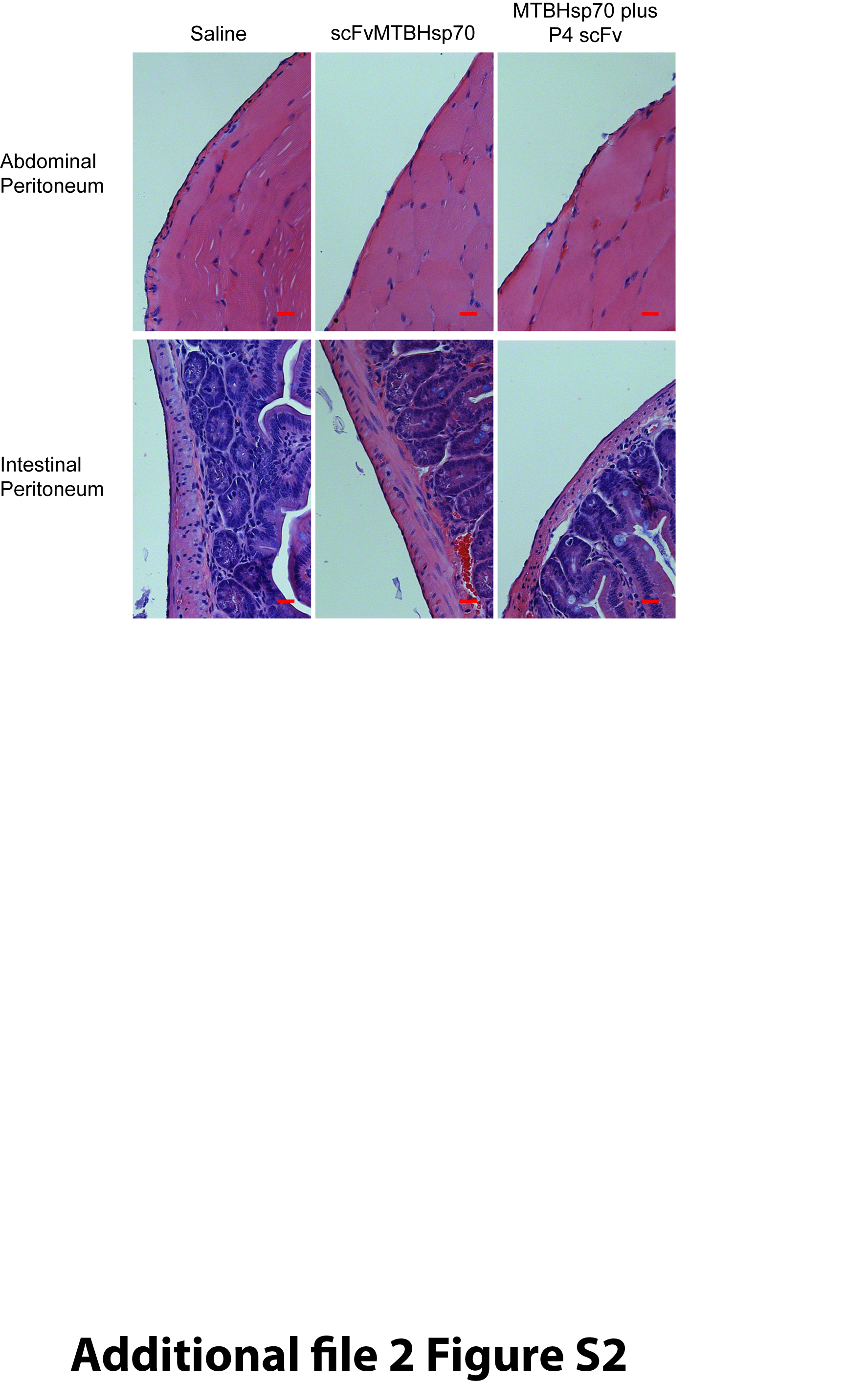

Supplement: Additional file 2: Figure S2 — scFvMTBHsp70 or MTBHsp70 plus P4 scFv treatment does not lead to infiltration of inflammatory cells into abdominal or intestinal mesothelial tissues. Samples of abdominal wall and intestine were prepared from C57BL/6 mice that had previously received multiple i.p. injections of scFvMTBHsp70, MTBHsp70 plus P4 scFv or saline as described in the Methods section. Sections of these tissues were stained with H&E, and images were acquired on a Zeiss Axio A1 microscope. Representative images from 3 animals per treatment group are shown. No detectable level of mononuclear cell or granulocyte infiltrate within mesothelial tissues was seen in any sampled tissues. Scale bar, 20 μm. [file 1756-8722-7-15-S2.tif]

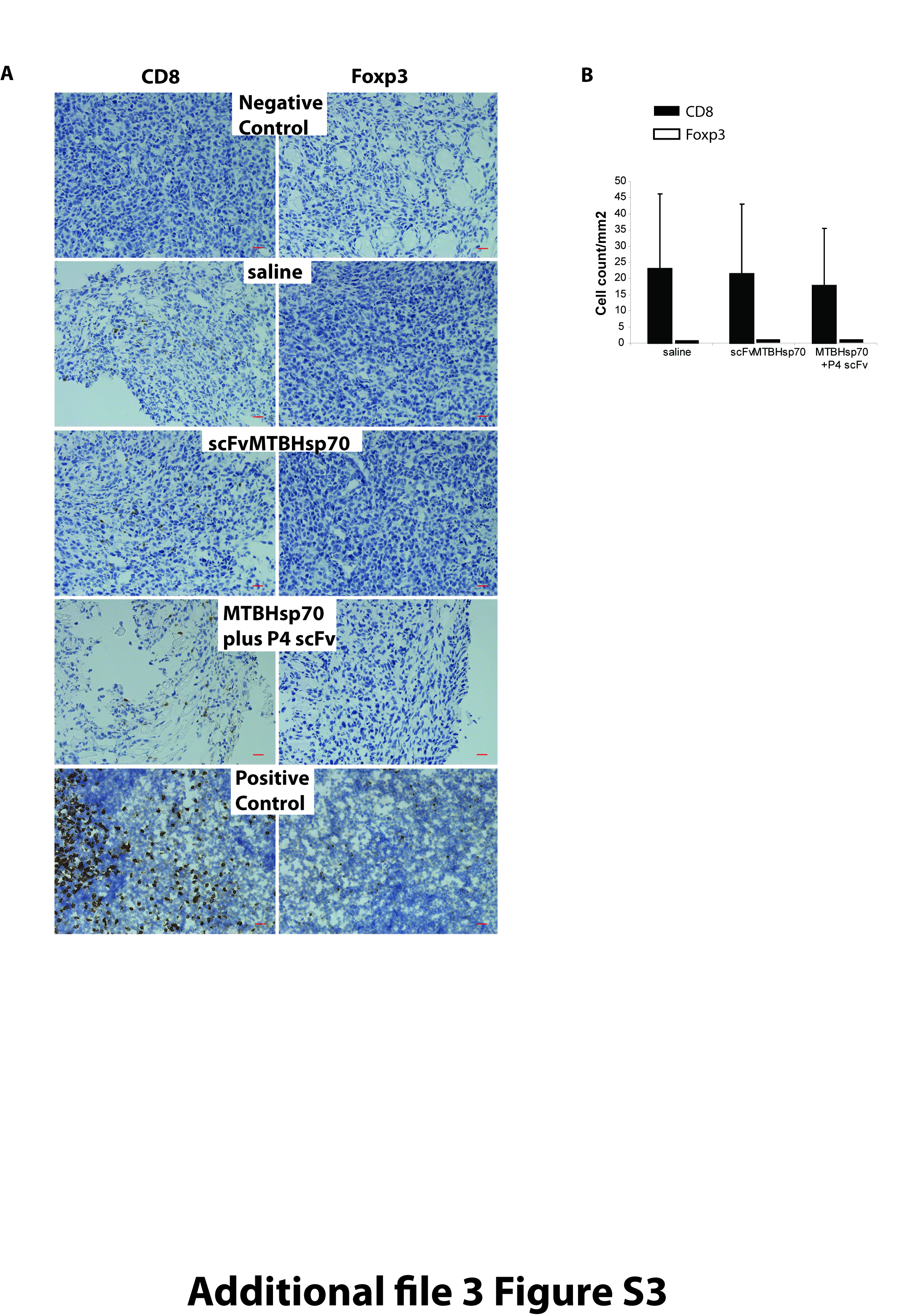

Supplement: Additional file 3: Figure S3 — scFvMTBHsp70 treatment does not affect numbers of tumor-infiltrating CD8+ or Foxp3+ T cells. (A) Representative images of intratumoral CD8+ and Foxp3+ T cells from saline (n = 3), scFvMTBHsp70 (n = 3), or MTBHsp70 plus P4 scFv (n = 3) -treated mice. Mouse spleen sections were used as positive controls: CD8+ and Foxp3+ T cells are clearly evident in the sections. Scale bar, 20 μm. (B) Numbers of CD8+ and Foxp3+ cells were quantified from 3–5 randomized fields. [file 1756-8722-7-15-S3.tif]

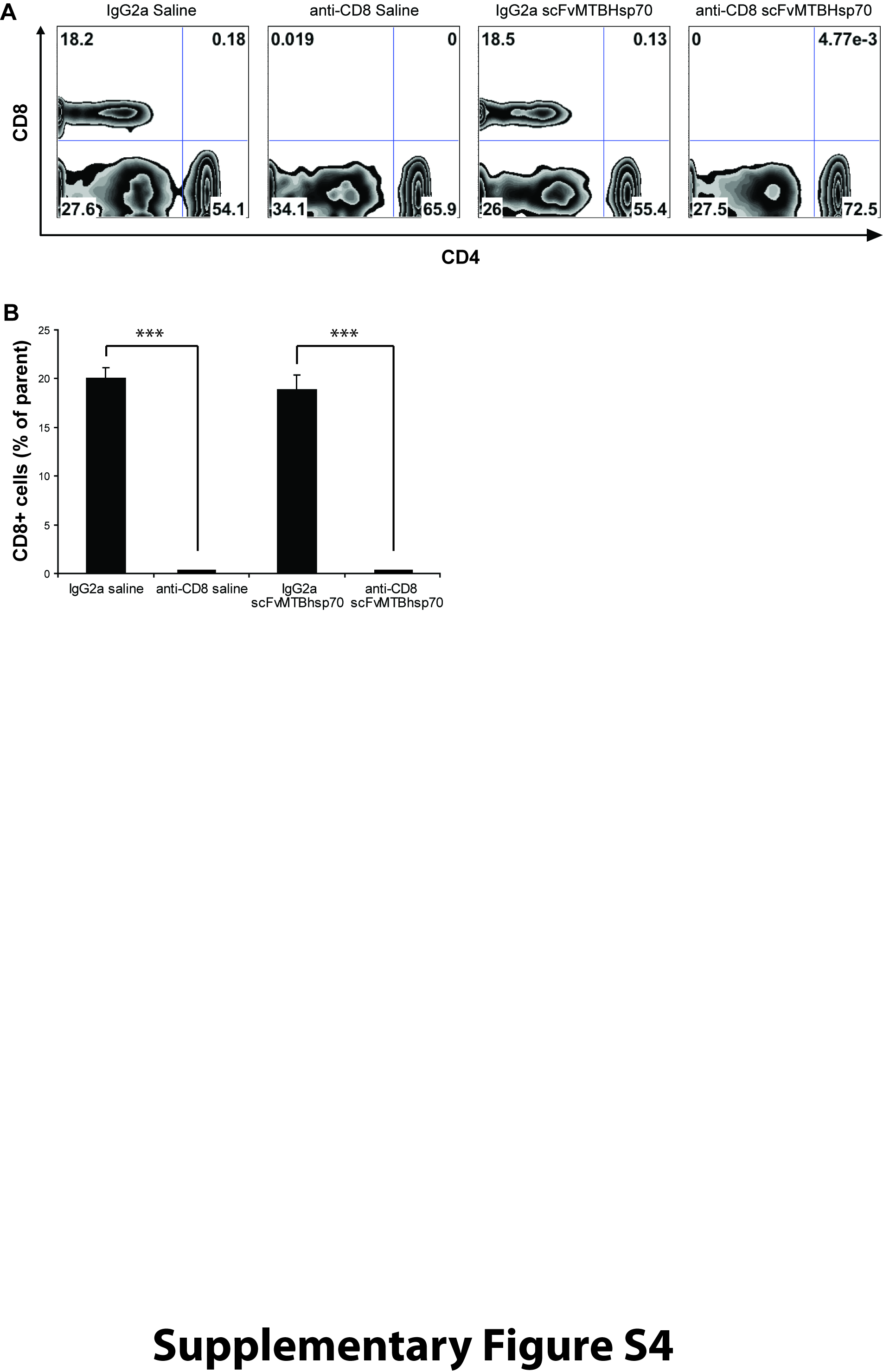

Supplement: Additional file 4: Figure S4 — Validation of in vivo depletion of CD8+ cells in FVB/NJ mice. Mice were injected i.p. with 200 μg of anti-CD8 mAb or an isotype-matched irrelevant rat IgG2a as described in Methods. All the mice were bled from the tail vein and the depletion of CD8+ cells was examined by flow cytometry analysis of peripheral blood cells stained with fluorophore-conjugated anti-CD8 on days 7 and 28 after tumor inoculation. (A) Representative results of flow analyses on 10 mice per group and reported as the percentage of CD8+ cells in lymphocytes. (B) CD8+ cells in the mice treated with isotype IgG2a or anti-CD8 mAb were compared. ***,p< 0.001. [file 1756-8722-7-15-S4.tiff]
